# Evaluating the use of different positional strategies for sentence selection in biomedical literature summarization

**DOI:** 10.1186/1471-2105-14-71

**Published:** 2013-02-27

**Authors:** Laura Plaza, Jorge Carrillo-de-Albornoz

**Affiliations:** 1 Universidad Autónoma de Madrid, C/Francisco Tomás y Valiente, 11, 28049 Madrid, Spain; 2 UNED NLP & IR Group, C/ Juan del Rosal, 16, 28040 Madrid, Spain

## Abstract

**Background:**

The position of a sentence in a document has been traditionally considered an indicator of the relevance of the sentence, and therefore it is frequently used by automatic summarization systems as an attribute for sentence selection. Sentences close to the beginning of the document are supposed to deal with the main topic and thus are selected for the summary. This criterion has shown to be very effective when summarizing some types of documents, such as news items. However, this property is not likely to be found in other types of documents, such as scientific articles, where other positional criteria may be preferred. The purpose of the present work is to study the utility of different positional strategies for biomedical literature summarization.

**Results:**

We have evaluated three different positional strategies: (1) awarding the sentences at the beginning of the document, (2) preferring those at the beginning and end of the document, and (3) weighting the sentences according to the section in which they appear. To this end, we have implemented two summarizers, one based on semantic graphs and the other based on concept frequencies, and evaluated the summaries they produce when combined with each of the positional strategies above using ROUGE metrics. Our results indicate that it is possible to improve the quality of the summaries by weighting the sentences according to the section in which they appear (≈17*%* improvement in ROUGE-2 for the graph-based summarizer and ≈20*%* for the frequency-based summarizer), and that the sections containing the more salient information are the *Methods and Material* and the *Discussion and Results* ones.

**Conclusions:**

It has been found that the use of traditional positional criteria that award sentences at the beginning and/or the end of the document are not helpful when summarizing scientific literature. In contrast, a more appropriate strategy is that which weights sentences according to the section in which they appear.

## Introduction and Motivation

The amount of biomedical literature being published is growing rapidly in recent years, making it difficult for researchers to find the information they need. In this context, text automatic techniques may help alleviate the information overload problem. First, automatic summaries may be useful in anticipating the contents of the original documents, so that users may decide which of the documents to read further. As stated in [1], even in the presence of the author’s abstract, there are two main reasons for wanting to generate text summaries from a full-text: (1) the abstract, which is usually limited to around 200 words, may be missing relevant content, and (2) there is not a single ideal summary, but rather, the ideal summary depends on the user’s information needs. Moreover, automatic summaries have been shown to improve indexing and categorization of biomedical literature when used as substitutes for the articles’ abstracts [2,3], since they help to filter non relevant and noisy information.

Text summarization refers to the process of generating a brief summary of one or several documents [4]. Summaries may be extractive or abstractive. Extractive summaries are created by identifying salient textual units (i.e., sentences or paragraphs) in the sources, while abstractive summaries are built by paraphrasing the information in the original documents. In other words, while extractive summarization is mainly concerned with what the summary content should be, abstractive summarization puts the emphasis on the form [5]. Although human summaries are typically abstracts, most existing systems produce extracts largely because extractive summarization has been demonstrated to report better results than abstractive summarization [6]. This is due to the difficulties that the abstraction process entails, which usually involves the identification of the most prevalent concepts in the source, the appropriate semantic representation of them, a minimum level of inference and the rewriting of the summary through Natural Language Generation techniques.

Extractive methods typically construct summaries based on a superficial analysis of the text. The most popular approaches include statistical techniques and graph-based methods (see [4,7] for a more detailed study of summarization techniques). Graph-based methods represent the text as a graph, where the nodes correspond to word, sentences or even concepts, and the edges represent various types of syntactic and semantic relations among them. Different clustering methods are then applied to identify salient nodes within the graph and to extract the sentences for the summary.

Statistical approaches are based on simple heuristics to rank the sentences for the summary, such as the position of the sentences in the document [8-12], the frequency of their terms [9,10,13-17], the presence of certain cue words [14,18,19], and the word overlap between the sentences and the document title and headings [11,14]. Despite their simplicity, these features are commonly used in the most recent works on extractive summarization, usually in combination with other more complex approaches, such as graph-based or template-based [20,21].

Focusing on the position of sentences in a document, this has been traditionally considered an important factor in finding the sentences that are most related to the central topic of the document, and used in different NLP tasks [8,22,23]. Baxendale [8] examined 200 paragraphs to find that in 85% of the paragraphs the topic sentence came as the first one and in 7% of the time it was the last sentence. Other works argue that the sentences close to the beginning and/or the end of the document are supposed to deal with the main theme of the document, and so more weight is assigned to them [11,12]. This criterion has showed to be very effective when summarizing some types of documents, such as news items, where the information is placed following the inverted pyramid structure (i.e., the most important information is placed first but, as the article continues, the less important details are presented) [24]. However, as stated in [5,22], even though texts generally follow a predictable discourse structure, and the sentences of greater topic centrality tend to occur in certain specifiable locations, this structure significantly varies over domains and the importance of the sentence position must be evaluated *ad hoc* for each domain and type of document.

Regarding summarization of biomedical literature, our previous work [20] showed that using a positional function that attaches greater relevance to sentences close to the beginning and end of the document together with a semantic graph-based summarization approach decreases performance compared with not using any positional information. This result was not surprising because scientific papers are not (a priori) expected to present the core information at the beginning and end of the document. In contrast, the first sentences in scientific papers usually introduce the motivation of the study, whereas the last sentences provide conclusions and future work. The most important information is expected to be found in the middle sentences, as part of the method, results, and discussion sections. Therefore, it seems that a more appropriate positional criterion would be one that gives priority to sentences belonging to such central sections. This intuition, however, needs to be empirically evaluated.

Following this idea, in the present work we study if the use of different positional criteria may be of help when summarizing scientific biomedical articles. In particular, three strategies are examined: (1) awarding the sentences at the beginning of the document, (2) preferring those at the beginning and end of the document, or (3) weighting the sentences according to the section (or section group) in which they appear. To this end, we have implemented two different summarizers, one based on semantic graphs and the other based on concept frequencies, and evaluated the summaries they produce when combined with each of the positional strategies above. Our results show that it is possible to improve the quality of the summaries that are generated by weighting the sentences according to the section in which they appear and giving priority to sentences from the *Method and Material* and *Results and Discussion* sections of the article. We believe these results to be of great interest since they may guide NLP tasks involving extraction of salient information in biomedical literature.

The paper is structured as follows. We first present some related work in biomedical summarization. We next describe several positional strategies for sentence selection, along with the two summarizers. We evaluate the summaries generated by both summarizers using the different positional strategies and present the evaluation results. These results are then discussed. We finally draw the main conclusions of the study and outline future work.

## Background

Even though the first works in automatic text summarization date from the middle of the last century [13], research in biomedical summarization has started only recently. Biomedical summarization works typically adapt existing methods from domain-independent summarization to deal with the highly specialized biomedical terminology. To this end, they make use of external knowledge sources to represent the texts as sets of domain concepts and relations. This produces a richer representation than the one provided by traditional term-based models and results in better quality summaries.

A pioneer work in biomedical summarization is found in [25]. They propose the use of semantic predications provided by SemRep [26] and information from the Unified Medical Language System (UMLS)^â“‡^[27] to extract biomedical entities and relations, and generate semantic-level abstracts, which are presented in graphical format. Ling et al. [28] focus on a narrower domain, genomic, and present a gene summary system that ranks sentences according to three features: the relevance of six gene aspects, such as the DNA sequence, the relevance of the documents where the sentences are taken from, and the position of the sentences in the document. Reeve et al. [1] use the frequency of the UMLS Metathesaurus concepts found in the text and adapt the lexical chaining approach [29] to deal with concepts instead of terms. Their system is used to produce single-document extracts of biomedical articles.

More sophisticated is the work of Yoo et al. [30] for multi-document summarization. They represent a corpus of documents as a graph, where the nodes are the MeSH^â“‡s^[31] descriptors found in the corpus and the edges represent hypernymy and co-occurrence relations between them. They cluster the MeSH concepts in the corpus to identify sets of documents dealing with the same topic and then generate a summary from each document cluster. BioSquash [32] is a question-oriented extractive system for biomedical multi-document summarization. It constructs a semantic graph that contains concepts of three types: ontological concepts (general ones from WordNet [33] and specific ones from the UMLS), named entities and noun phrases.

More recent is the work of Shang et al. [34], where the aim is to combine information retrieval techniques with information extraction methods to generate text summaries of sets of documents describing a certain topic. To do this, they use SemRep to extract relations among UMLS Metathesaurus concepts and a relation-level retrieval method to select the relations more relevant to a given query concept. Finally, they extract the most relevant sentences for each topic based on the previous ranking of relations and the location of the sentences in different sections of the document. However, no details are given about how the location scores are calculated.

## Methods

In this section, we first present the different positional strategies for sentence selections. We next describe the two different summarizers (one based on semantic graphs and the other based on concept frequencies) that have been developed to test such positional strategies.

### Positional strategies for sentence selection

In order to test our hypothesis that the position of a sentence in the different sections of the document is an indication of the importance of the sentence for inclusion in a summary, and that traditional positional strategies are not appropriate for summarizing biomedical literature, we have defined the following positional criteria: 

•**Distance to the beginning (*Begin-Pos*):** Sentences close to the beginning of the document are supposed to the deal with the central topic of the document, and so more weight is assigned to them. Thus, a score *Begin*_*Pos*(*S*_*j*_)∈(0,1] is calculated as the reciprocal to the position of the sentence in the document, as shown in equation 1, where *m*_*j*_ represents the position of the sentence, *S*_*j*_, within the document. In this way, for instance, for the first sentence in the document $\text{Begin}\text{\_}\text{Pos}(S_{1})=\frac{1}{1}=1$, for the second sentence $\text{Begin}\text{\_}\text{Pos}(S_{2})=\frac{1}{2}=0.5$, and so on.

(1) $$\text{Begin}\text{\_}\text{Pos}(S_{j})=\frac{1}{m_{j}}$$

•**Distance to the beginning and end (*Begin-End-Pos)*:** Sentences close to the beginning and the end of the document are considered highly relevant. Therefore, a score *Begin*_*End*_*Pos*(*S*_*j*_)∈(0,1] is calculated for each sentence as shown in equation 2, where *M* represents the number of sentences in the document and *m*_*j*_ represents the position of the sentence, *S*_*j*_, within the document. This equation returns the maximum of the reciprocal to the position of the sentence from the beginning and the end of the document. In this way, for instance, if M=10, then for the first sentence in the document $\text{Begin}\text{\_}\text{End}\text{\_}\text{Pos}(S_{1})=\text{max}\{\frac{1}{1},\frac{1}{10-1+1}\}=1$, for the second sentence $\text{Begin}\text{\_}\text{End}\text{\_}\text{Pos}(S_{2})=\text{max}\{\frac{1}{2},\frac{1}{10-2+1}\}=\frac{1}{2}=0.5$, and for the last sentence $\text{Begin}\text{\_}\text{End}\text{\_}\text{Pos}(S_{10})=\text{max}\{\frac{1}{10},\frac{1}{10-10+1}\}=1$.

(2) $$\text{Begin}\text{\_}\text{End}\text{\_}\text{Pos}(S_{j})=\text{max}\left\{\frac{1}{m_{j}},\frac{1}{M-m_{j}+1}\right\}$$

•**Section in the document (*Section-Pos*):** We consider the following section classes or clusters: *(1) Introduction*, *(2) Background*, *(3) Materials and Methods*, *(4) Results and Discussion*, and *(5) Conclusions and Future Work*. Following the methodology used in [2], we have first analyzed the typical structure of biomedical articles and manually grouped the section headers in these five section groups based on the similarity of their contents. Lexical variants of the same section header are included in the same class. For example, *Method* and *Methods* are clustered in the *Materials and Methods* class. Similarly, sections that differ in their title but refer to the same content are included in the same class (e.g., *Experimental Procedures* is also included in the class *Material and Methods*). We calculate a score *Section*_*Pos*(*S*_*j*_)∈(0,1] according to the equation 3, where the functions *Intro*(*S*_*j*_), *BackG*(*S*_*j*_), *M*&*M*(*S*_*j*_), *R*&*D*(*S*_*j*_), and *C*&*F*(*S*_*j*_) are equal to 1 if the sentence *S*_*j*_ belongs to each of the five section groups, respectively, and 0 otherwise. The values of *γ*, *δ*, *θ*, *σ*, and *Π* vary between 0 and 1, and need to be empirically determined.

(3) $$\begin{array}{ll} \text{Section\_}\text{Pos}(S_{j})= & \gamma \times \text{Intro}(S_{j})+\delta \times \text{BackG}(S_{j})\\ & +\theta \times M\&M(S_{j})+\sigma \times R\&D(S_{j})\\ & +\Pi \times C\&F(S_{j}) \end{array}$$

### Graph-based summarizer

**Figure 1:**
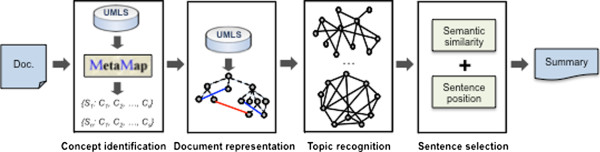
**Architecture of the graph-based summarization system.** The figure illustrates the different steps in the summarization process: (i) concept identification, (ii) document representation, (iii) topic recognition and (iv) sentence selection.

We use the graph-based summarization method presented in [20]. This method is based on the representation of the document as a conceptual graph, using the UMLS [27] as the knowledge source, and the use of a degree-based clustering algorithm for detecting salient concepts within the graph. The original summarizer has been modified to incorporate more advanced positional strategies in the sentence selection step. The system architecture is illustrated in Figure 1.

The method consists of the 4 main steps, which are briefly explained below (see [20] for a detailed explanation): 

•The first step, **concept identification**, is to map the document to concepts from the UMLS Metathesaurus and semantic types from the UMLS Semantic Network. We run the MetaMap [35] program over the body section of the document to obtain the Metathesaurus concepts that are found within the text. MetaMap is invoked using the word sense disambiguation option (-y flag). This flag implements the Journal Descriptor Indexing (JDI) methodology described in [36]. UMLS concepts belonging to very general semantic types are discarded since they have been found to be excessively broad and do not contribute to summarization. These types are *Quantitative concept*, *Qualitative concept*, *Temporal concept*, *Functional concept*, *Idea or concept*, *Intellectual product*, *Mental process*, *Spatial concept* and *Language*.

•The second step, **document representation**, is to construct a graph-based representation of the document. To do this, we first extend the UMLS concepts with their complete hierarchy of hypernyms (*is_a* relations) and merge the hierarchies of all the concepts in the same sentence to construct a *sentence graph*. The upper levels of these hierarchies are removed, since they represent concepts with excessively broad meanings. Next, all the sentence graphs are merged into a single *document graph*. This graph is extended with two further types of relations: relations between concepts in the UMLS Metathesaurus and relations between semantic types in the UMLS Semantic Network (see [37] for a description of the different relationships in the UMLS). Finally, each edge is assigned a weight in [0, 1] as shown in equation 4. The weight of an edge *e* representing an *is_a* relation between two vertices, *v*_*i*_ and *v*_*j*_ (where *v*_*i*_ is a parent of *v*_*j*_), is calculated as the ratio of the depth of *v*_*i*_ to the depth of *v*_*j*_ from the root of their hierarchy. The weight of an edge representing any other relation (i.e. *associated with* and *other related*) between pairs of leaf vertices is always 1.

(4) $$\text{weight}(e,v_{i},v_{j})=\beta$$

$$\text{where}\left\{\begin{array}{ll} \beta =\frac{\text{depth}(v_{i})}{\text{depth}(v_{j})} & \;\text{if}\;e\;\text{represents an}\;\text{is}\text{\_}a\;\text{relation}\\ \beta =1 & \;\text{otherwise} \end{array}\right)$$

**Figure 2:**
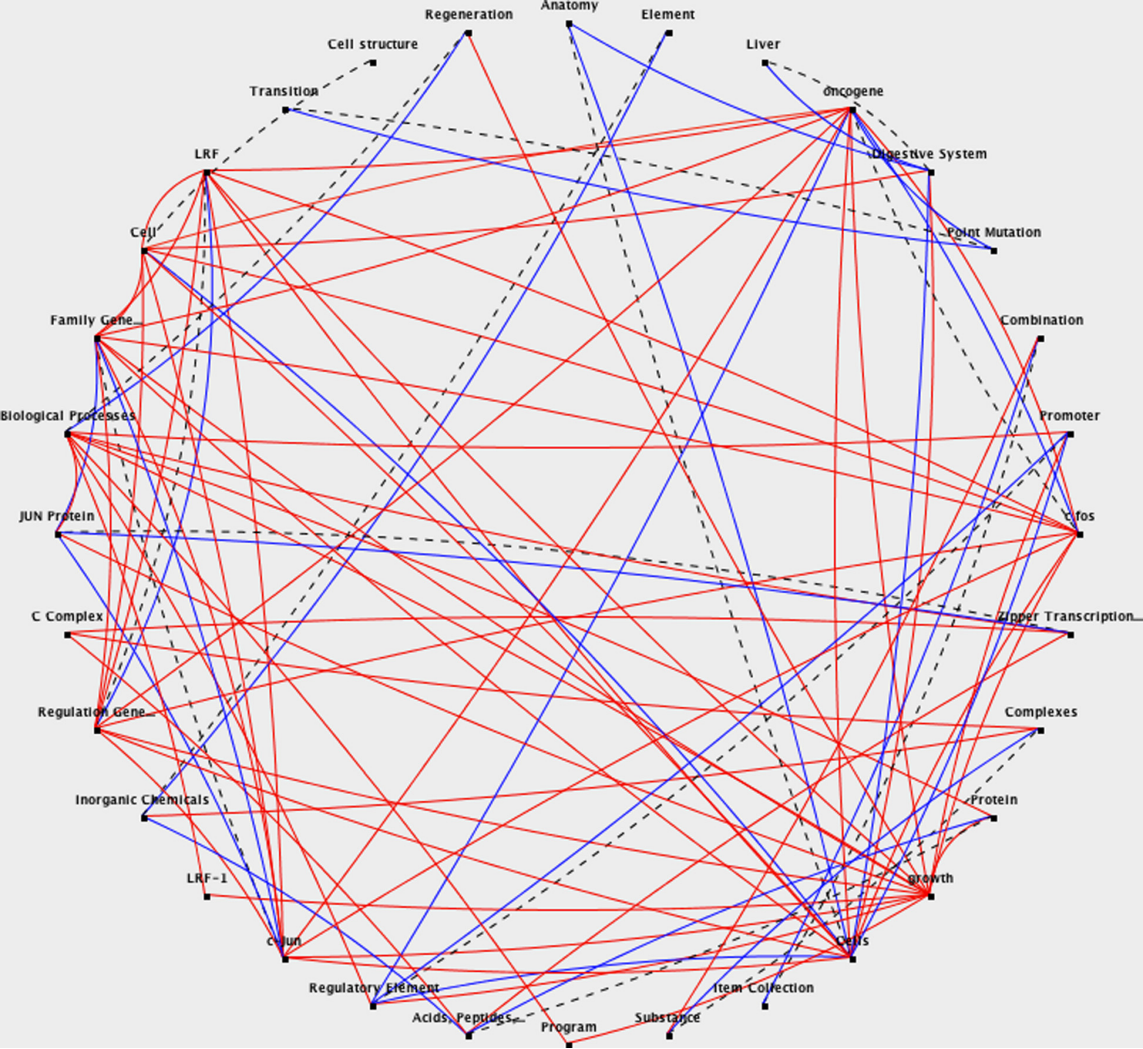
**Example document graph.** Dashed black lines represent hypernymy relations; red lines represent Metathesaurus relations; and blue lines represent Semantic Network relations.

•To illustrate this process, Figure 2 shows the document graph for the following text from [38]: 

Interactions among LRF-1, JunB, c-Jun, and c-Fos define a regulatory program in the G1 phase of liver regeneration. In regenerating liver, a physiologically normal model of cell growth, LRF-1, JunB, c-Jun, and c-Fos among Jun/Fos/LRF-1 family members are induced posthepatectomy. In liver cells, high levels of c-Fos/c-Jun, c-Fos/JunB, LRF-1/c-Jun, and LRF-1/JunB complexes are present for several hours after the G0/G1 transition, and the relative level of LRF-1/JunB complexes increases during G1. We provide evidence for dramatic differences in promoter-specific activation by LRF-1- and c-Fos-containing complexes. LRF-1 in combination with either Jun protein strongly activates a cyclic AMP response element-containing promoter which c-Fos/Jun does not activate.

•The third step, **topic recognition**, consists of clustering the UMLS concepts in the document graph using a degree-based clustering method similar to that used by [30]. The aim is to construct sets of concepts strongly related in meaning, based on the assumption that each of these clusters represents a different topic in the document. We first compute the *salience* of each vertex in the graph, as the sum of the weights of the edges that are linked to it. Next, the nodes are ranked according to its salience. The *n* vertices with a highest salience are labeled as *hub vertices*. The clustering algorithm then groups the hub vertices into *hub vertex sets* (HVS). These can be interpreted as sets of concepts strongly connected and will represent the centroids of the final clusters. The remaining vertices (i.e. those not included in the HVS) are iteratively assigned to the cluster to which they are more connected. The output of this step is, therefore, a number of clusters of UMLS concepts, each cluster represented by the set of most highly connected concepts within it (the so-called HVS). In this way, for instance, the top five salient concepts in the document graph represented in Figure 2 are: *cells*, *LRF*, *cJUN*, *c-fos*, and *growth*.

•The last step, **sentence selection**, consists of computing the similarity between each sentence graph and each cluster, and selecting the sentences for the summary based on these similarities. To compute sentence-to-cluster similarity, we use a non-democratic vote mechanism so that each vertex of a sentence assigns a vote to a cluster if the vertex belongs to its HVS, half a vote if the vertex belongs to it but not to its HVS, and no votes otherwise. The similarity between the sentence graph and the cluster is computed as the sum of the votes assigned by all the vertices in the sentence graph to the cluster. A single score for each sentence is calculated, as the sum of its similarity to each cluster adjusted to the cluster’s size (equation 5).

(5) $$\text{Sem}\text{\_}\text{Sim}(S_{j})=\underset{C_{i}}{\sum }\frac{\text{similarity}(C_{i},S_{j})}{\left|C_{i}\right|}$$

Finally, this semantic similarity is normalized in the [0,1] interval and combined with each of the positional criteria explained in the previous section using a linear function (see equation 6). The N sentences with higher score are then selected for the summary.

(6) $$\text{Score}(S_{j})=\alpha \times \text{Sem}\text{\_}\text{Sim}(S_{j})+\beta \times \text{Position}(S_{j});$$

*α* and *β* can be assigned different weights between 0 and 1. Their optimal values must be determined empirically.

### Concept frequency-based summarizer

The second summarizer is based on the frequency of UMLS concepts in the document. It consists of 4 steps: 

•The first step, **concept identification**, is to map the document to concepts from the UMLS Metathesaurus and semantic types from the UMLS Semantic Network using MetaMap, as explained for the graph-based summarizer.

•**Concept frequency representation:** Following Luhn’s theory, we assume that the more times a word (or concept) appears in a document, the more relevant become the sentences that contain this word. In this way, if {*C*_1_,*C*_2_,...,*C*_*n*_} is the set of *n* Metathesaurus concepts that appear in the document *d*, and *f*_*i*_(*d*) is the number of times that *C*_*i*_ appears in it, then the document may be represented by the vector *D*={*f*_1_(*d*),*f*_2_(*d*),...,*f*_*n*_(*d*)}. Similarly, we build the vectors representing each sentence in the document, *S*_*j*_, and compute a concept frequency score, *CF*(*S*_*j*_), as the sum of the frequency of all the concepts in the sentence multiplied by the frequency of those concepts in the document. This *CF*(*S*_*j*_) score is normalized in the [0,1] interval.

•In this way, the text modeled as a conceptual graph in Figure 2 is represented by the following document and sentence vectors:

$$\begin{array}{lll} & D=\{\text{LRF}=7,c-\text{Jun}=3,c-\text{fos}=3,\text{Liver}=3,\text{Cell}=2,\text{Promoter}=2,\text{Complexes}=2,\; & \;\\ & \text{Program}=1,\text{Regeneration}=1,\text{growth}=1,\text{LRF}-1=1,\text{Transition}=1,C-\text{Complex}=1,\; & \;\\ & \text{Combination}=1,\text{JUNProtein}=1,\text{Protein}=1,\text{Element}=1\}\; & \;\\ & S_{1}=\{\text{LRF}=1,c-\text{Jun}=1,c-\text{fos}=1,\text{Program}=1,\text{Liver}=1,\text{Regeneration}=1\}\; & \;\\ & S_{2}=\{\text{LRF}=2,c-\text{Jun}=1,c-\text{fos}=1,\text{Liver}=1,\text{Cell}=1,\text{growth}=1\}\; & \;\\ & S_{3}=\{\text{LRF}=2,c-\text{Jun}=1,c-\text{fos}=1,\text{Liver}=1,\text{Cell}=1,\text{LRF}-1=1,\text{Transition}=1,\text{Complexes}=2\}\; & \;\\ & S_{4}=\{\text{LRF}=1,\text{Promoter}=1,C-\text{Complex}=1\}\; & \;\\ & S_{5}=\{\text{LRF}=1,\text{Promoter}=1,\text{Combination}=1,\text{JUNProtein}=1,\text{Protein}=1,\text{Element}=1\}\; & \;\\ & \; \end{array}$$

•The concept frequency scores of the sentences are, respectively, *CF*(*S*_1_)=18, *CF*(*S*_2_)=26, *CF*(*S*_3_)=31, *CF*(*S*_4_)=10 and *CF*(*S*_5_)=13.

•**Sentence position:** Different position scores are calculated for each sentence in the document, according to the positional strategies described previously in the article.

•The last step, **sentence selection**, consists of extracting the most important sentences for the summary up to the desired length. Having computed the different weights for each sentence, the final score for a sentence (*Score*(*S*_*j*_)) is calculated according to the equation 7. Finally, the *N* sentences with higher score are extracted for the summary.

(7) $$\text{Score}(S_{j})=\alpha \times \text{CF}(S_{j})+\beta \times \text{Position}(S_{j})$$

•*α* and *β* can be assigned different weights between 0 and 1. Their optimal values must be determined empirically.

### Evaluation methods

The most common approach to evaluating automatically generated summaries of a document (also known as *peers*) is to compare them against manually-created summaries (called *reference* or *model* summaries) and measure the similarity between their content. The more content that is shared between the peer and reference summaries, the better the peer summary is assumed to be. To the authors’ knowledge, no corpus of model summaries exists for biomedical articles. For this reason, in this work we use a collection of 100 biomedical scientific articles randomly selected from the PMC Open Access Subset [39]. When collecting the articles, we made sure that they present, at least, the five main following sections: *Introduction*, *Background*, *Methods*, *Results and Discussion*, and *Conclusions and Future Work*. The abstracts for the articles are used as model summaries, since they condensate the most relevant content in the articles and have been written manually.

The ROUGE metrics [40] are used to quantify the content similarity between the automatic summaries and the reference ones. ROUGE is a commonly used evaluation method for summarization which uses the proportion of n-grams between a peer and one or more reference summaries to compute a value within [0,1]. Higher values of ROUGE are preferred, since they indicate a greater content overlap between the peer and the model. The following ROUGE metrics are used: ROUGE-2 and ROUGE-SU4. ROUGE-2 counts the number of bigrams that are shared by the peer and reference summaries and computes a recall-related measure. Similarly, ROUGE-SU4 measures the overlap of skip-bigrams (i.e., pairs of words in their sentence order, allowing for arbitrary gaps), using a skip distance of 4. Both ROUGE-2 and ROUGE-SU4 have shown high correlation with the human judges gathered from the Document Understanding Conferences [41]. However, it must be noted that ROUGE metrics present two important limitations: (1) they depend on the length of the peer summaries (i.e., the longer is the peer with respect to the model, the higher are expected to be the ROUGE scores), and (2) since they use lexical matching instead of semantic matching, peer summaries that are worded different but have the same semantic information may be assigned different ROUGE scores. Thus, these metrics should only be used in a comparative fashion on the same dataset and should not be interpreted as absolute measures.

Automatic summaries are generated by selecting sentences until each summary reaches the same number of sentences than its corresponding model summary (i.e., the article’s abstract). We generate summaries using both the graph-based and the frequency-based summarizers and the three positional strategies for sentence selection, and assigning different weights to the different parameters of the summarizers. For these experiments, different combinations of values for *α*, *β*, *γ*, *δ*, *θ*, *σ*, and *Π* were tested. However, for the sake of brevity, only the combinations that produced the best ROUGE scores are presented. A Wilcoxon Signed Ranks Test with a 95% confidence interval is used to test statistical significance of the results.

## Results

We first evaluate the adequacy of the *Begin-Pos* positional strategy. The results of evaluating the automatic summaries generated when different weights are assigned to such criterion are shown in Table 1. As it may seen from this table, giving greater weight to sentences at the beginning of the document improves the quality of the automatic summaries compared with not using positional information, but only when the weight assigned to the position of the sentences is low (from *0.1* to *0.2*). The improvement achieved is higher for the frequency-based summarizer than for the graph-based one, and it is only statistically significant for the first one.

**Table 1:** **ROUGE scores for the summaries generated using the *Begin-Pos *strategy**

**Weights**		**Graph-based**		**Frequency-based**	
***α***	***β***	**R-2**	**R-SU4**	**R-2**	**R-SU4**
1.0	0.0	0,1660	0,1334	0,1375	0,1096
0.9	0.1	**0,1744**	**0,1492**	0,1502	0,1223
0.8	0.2	0,1668	0,1357	0,1574*	0,1290*
0.75	0.25	0,1592	0,1315	0,1511	0,1205

Significance is calculated with respect to the non-positional information baseline (***β=0.0***), and shown using the following convention: * = p <.05 and no star indicates non-significance. The best scores per summarizer are shown in bold.

We next evaluate the effect of the *Begin-End-Pos* criterion, which attaches greater weight to sentences close to the beginning and end of the document. It may be seen from Table 2 that this strategy does not benefit the quality of the graph-based summaries, regardless of the weights assigned to the different criteria, but slightly improves the performance of the concept-frequency based summarizer when *β* is set to *0.1*. However, once again, the improvement achieved is not significant.

**Table 2:** **ROUGE scores for the summaries generated using the *Begin-End-Pos *strategy**

**Weights**		**Graph-based**		**Frequency-based**	
***α***	***β***	**R-2**	**R-SU4**	**R-2**	**R-SU4**
1.0	0.0	**0,1660**	**0,1334**	0,1375	0,1096
0.9	0.1	0,1610	0,1305	**0,1503**	**0,1223**
0.8	0.2	0,1572	0,1298	0,1498	0,1220
0.75	0.25	0,1546	0,1259	0,1436	0,1182

Significance is calculated with respect to the non-positional information baseline (***β=0.0***), and shown using the following convention: * = p <.05 and no star indicates non-significance. The best scores per summarizer are shown in bold.

We finally examine the summaries generated when different weights are assigned to sentences depending on the section in which they appear (*Section-Pos* strategy). These results are shown in Table 3. For both summarizers, there exist a combination of weights that produces significantly better summaries compared with the non-positional information summaries (*β*=0.0). In particular, the best ROUGE scores are reported when the *Introduction* and the *Conclusions and Future Work* sections are given a weight of *0.2*, no weight is assigned to sentences from the *Related Work* section, and the sentences from the *Methods and Material* and the *Results and Discussion* sections are given a weight of *1.0*. This configuration allows for improvements of over 17% in ROUGE-2 for the graph-based summarizer and over 20% for the frequency-based summarizer.

**Table 3:** **ROUGE scores for the summaries generated using the *Section-Pos *strategy**

**Weights**							**Graph-based**		**Frequency-based**	
***α***	***β***	***γ***	***δ***	***θ***	***σ***	***Π***	**R-2**	**R-SU4**	**R-2**	**R-SU4**
1.0	0.0	-	-	-	-	-	0,1660	0,1334	0,1375	0,1096
		0.2	0.1	1.0	0.4	0.2	0,1635	0,1341	0,1395	0,1134
0.9	0.1	0.2	0.0	0.8	0.6	0.1	0,1752	0,1483	0,1402	0,1159
		0.2	0.0	1.0	1.0	0.1	0,1811*	0,1492*	*0,1461*	*0,1186*
		0.2	0.0	1.0	0.8	0.0	0,1758	0,1490	0,1423	0,1178
		0.2	0.1	1.0	0.4	0.2	0,1726	0,1489	0,1546	0,13254
0.8	0.2	0.2	0.0	0.8	0.6	0.1	0,1758	0,1514	0,1589	0,1332
		0.2	0.0	1.0	1.0	0.1	0,1951*	0,1610*	0,1653*	0,1352*
		0.2	0.0	1.0	0.8	0.0	0,1846*	0,1526*	0,1610*	0,1314*
		0.2	0.1	1.0	0.4	0.2	0,1613	0,1333	0,1583*	0,1298*
0.75	0.25	0.2	0.0	0.8	0.6	0.1	0,1634	0,1348	0,1598*	0,1302*
		0.2	0.0	1.0	1.0	0.1	*0,1752*	*0,1499*	0,1615*	0,1307*
		0.2	0.0	1.0	0.8	0.0	0,1688	0,1353	0,1604	0,1306

Significance is calculated with respect to the non-positional information baseline (***β=0.0***), and shown using the following convention: * = p <.05 and no star indicates non-significance. The best scores per summarizer are shown in bold.

It is also worth mentioning that the experiments showed that weights for the *Background* and *Conclusion and Future Work* sections (i.e., *δ* and *Π* values) above *0.1* produce very poor summarization results, and that *γ* values (i.e., the weight for the *Introduction* section) upper *0.2* decrease performance as well. In contrast, the best results are reported when the *Methods and Material* and the *Results and Discussion* sections are assigned high weights.

Finally, Table 4 compiles the best results for each positional strategy and summarizer. For comparison purposes, this table also shows the ROUGE scores for the summaries generated using LexRank [42]. LexRank is the best-known graph-based method for summarization. It models documents as undirected graphs in which each node corresponds to a sentence, represented by its TF-IDF vector, and the edges are labeled with the cosine similarity between the sentences. It may be seen from Table 4 that the best ROUGE scores are obtained when the graph-based summarizer is combined with information about the position of the sentences in the different document sections. These scores are significantly better than those of the frequency-based summarizer and than those of LexRank.

**Table 4:** Comparison of summarization approaches

**Summarizer**	**ROUGE-2**	**ROUGE-SU4**
Graph-based	0,1660	0,1334
Graph-based + *Begin-Pos*	0,1744	0,1492
Graph-based + *Begin-End-Pos*	0,1610	0,1305
Graph-based + *Section-Pos*	**0,1951**	**0,1610**
Frequency-based	0,1375	0,1096
Frequency-based + *Begin-Pos*	0,1574	0,1290
Frequency-based + *Begin-End-Pos*	0,1503	0,1223
Frequency-based + *Section-Pos*	**0,1653**	0,1352
LexRank	**0,1628**	**0,1346**

ROUGE results for different summarization approaches. The best scores per summarizer are shown in bold.

## Discussion

The results in Tables 1, 2, and 3 confirm our hypothesis that traditional positional strategies are not appropriate when summarizing biomedical scientific articles, as opposed to summarizing other types of documents, such as news items. Our experiments have shown that awarding sentences close to the end of the document decreases summarization performance comparing with not using any positional information, while awarding sentences close to the beginning of the document only improves the quality of the summaries for the frequency-based summarizer.

In contrast, we have found that it is possible to improve summarization by taking into account the section in the article in which the different sentences appear, and attaching greater relevance to sentences from the appropriate sections. In particular, it has been found that sentences from the *Methods and Material* and *Results and Discussion* sections are more relevant for inclusion in the summary, since they are more related to the main topic of the document that sentences in other sections, such as *Introduction* and *Conclusions and Future Work*. Sentences in the *Related Work* section seem to be secondary, and therefore are not usually included in a summary. These results confirm what may be observed in the abstracts of the articles. If we examine such abstracts we realize that the information considered as important by the authors of the articles is mostly related to the method and results being described in the article, while the remaining sections are given less credit.

An interesting finding is that, in general, the frequency-based approach takes more advantage from the information about the position of sentences in the document than the graph-based one. We think this is due to the fact that the use of the frequency of the concepts alone is not enough to capture the salience of the sentences, and so the use of the positional criteria helps to bias the selection of sentences toward the most relevant information.

In contrast, the graph-based method captures better the importance of the different sentences, and thus produces better quality summaries even when no positional information is used. However, it still presents some limitations that will be addressed in future work. The first limitation is to do with the coverage of the UMLS. While general clinical terms are quite well covered, other vocabulary, specially that related to genomic, is not well supported [43]. As a results, automatic summaries of genetic and proteomic articles present low ROUGE values.

The second limitation is to do with the accuracy of the MetaMap mappings and the ambiguity in the UMLS Metathesaurus. Even using the -y disambiguation option, the precision of the JDI algorithm is reported to be around 0.78 when evaluated against a set of 45 ambiguous terms from the NLM-WSD corpus [44]. This precision, however, is expected to be lower for genomic entities, such as protein and gene names, where ambiguity is more frequent.

Third, our summarization and evaluation methods assume that all users have the same information needs, and that these needs are reflected in the authors’ abstracts. However, different users may have different interests. In future work, we plant to extend the summarizer to produce query-based summaries that take into account the readers’ information needs as specified in a user’s query. To this end, the similarity of each sentence in the document to the user’s query may be computed and uses as a feature for sentence selection.

## Conclusions

This work explores the utility of the position of the sentences as a feature for automatic summarization of scientific articles. Toward this goal, we have developed two different summarizers, one based on semantic graphs and the other using concept frequencies, which implement three different positional strategies: the first gives more importance to sentences at the beginning of the article, the second prefers sentences both at the beginning and end, and the third weights sentences according to the section in which they appear. The summaries generated are evaluated and compared with non-positional summaries.

Overall, the results suggest that it is possible to improve summarization by taking into account the section in the article in which the different sentences appear, and attaching greater relevance to sentences from the appropriate sections. In contrast, traditional strategies that attach greater weights to sentences at the beginning and end of the document are not suitable when summarizing biomedical scientific articles. We believe that our results are of great interest since they may guide NLP tasks involving extraction of salient information in biomedical literature.

As future work, we plan to investigate the importance of the specific location of sentences within the different sections of the article. In this way, for instance, the last sentences of the *Introduction* section may be more relevant that the first sentences in the same sections, since they usually anticipate the content of the document.

## Competing interests

The authors declare that they have no competing interests.

## Authors’ contributions

LP designed and developed the graph-based method for automatic summarization and performed the evaluation. JCdA designed and developed the concept frequency-based summarizer and performed the evaluation. LP and JCdA drafted and reviewed the manuscript. Both authors read and approved the final manuscript.
